# Effects of short-term travel on COVID-19 spread: A novel SEIR model and case study in Minnesota

**DOI:** 10.1371/journal.pone.0245919

**Published:** 2021-01-22

**Authors:** Michael W. Levin, Mingfeng Shang, Raphael Stern

**Affiliations:** Department of Civil, Environmental, and Geo- Engineering, University of Minnesota, Minneapolis, Minnesota, United States of America; Universitatsmedizin Greifswald, GERMANY

## Abstract

The novel coronavirus responsible for COVID-19 was first identified in Hubei Province, China in December, 2019. Within a matter of months the virus had spread and become a global pandemic. In addition to international air travel, local travel (e.g. by passenger car) contributes to the geographic spread of COVID-19. We modify the common susceptible-exposed-infectious-removed (SEIR) virus spread model and investigate the extent to which short-term travel associated with driving influences the spread of the virus. We consider the case study of the US state of Minnesota, and calibrated the proposed model with travel and viral spread data. Using our modified SEIR model that considers local short-term travel, we are able to better explain the virus spread than using the long-term travel SEIR model. Short-term travel associated with driving is predicted to be a significant contributor to the historical and future spread of COVID-19. The calibrated model also predicts the proportion of infections that were detected. We find that if driving trips remain at current levels, a substantial increase in COVID-19 cases may be observed in Minnesota, while decreasing intrastate travel could help contain the virus spread.

## Introduction

In December, 2019, a novel coronavirus (SARS-CoV-2), that causes the COVID-19 disease, was first identified in Hubei Provence, China. The virus quickly spread throughout China and, within several weeks, cases were being reported across Asia. By mid March, 2020, the World Health Organization (WHO) officially declared COVID-19 a global pandemic [1]. While many transmission cases are from direct contact within the community [2–4], one factor that may facilitate this human-to-human transmission is the underlying transportation network, which acts to propagate the virus between communities. While there is indication that travel is one mechanism by which the virus has spread globally [5–8], the extent to which local travel influences the spread of the virus is yet unknown. Specifically, while long-distance travel (e.g., by airplane) may help spread the virus over long distances, local travel (e.g., by passenger vehicle) may help accelerate the spread of the virus in a smaller geographic area. By “local” or “short-term” travel, we refer to an infectious person traveling from city *i* to city *j*, infecting others at *j*, then soon thereafter returning to *i* (as opposed to remaining in *j* for treatment).

**Fig 1:**
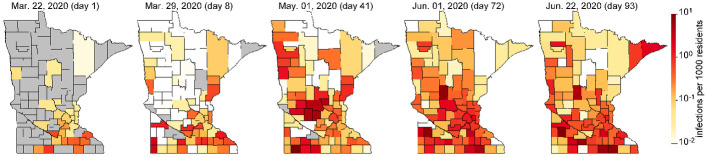
Evolution of COVID-19 infections in Minnesota (see S1 Video for more detail).

To study the extent to which local travel influences the spread of COVID-19, we modify the commonly used *susceptible-exposed-infectious-removed* (SEIR) [9] to account for increased transmission risk associated with short-term travel that may result from intrastate travel. These trips differ from long-distance travel since the travelers may only spend a short time at their destination, and will likely return to their home by the end of the day or within a day or two. The augmented model is demonstrated on viral spread data collected for the 87 counties in the US State of Minnesota (with population 5,639,589) between March 22 and June 22, 2020 and is calibrated with passenger car transportation data obtained during the same time period that tells the number of individual vehicles driving between counties in the entire state. Like many other regions, Minnesota is characterized by having one main international airport in Minneapolis/St. Paul with only smaller regional airports in other cities. Much of the state is rural. Therefore, although the introduction of COVID-19 into Minneapolis/St. Paul can be explained through international travel, much of its spread around the state may be more attributed to short-term road vehicle travel (see spread of COVID-19 in Minnesota in Fig 1 and S1 Video. Minnesota is therefore an ideal case study as well as being representative of short-term travel behavior in many other regions.

The SEIR model has been widely used to describe viral spread [10–12], including COVID-19 [13–16]. In the model, every member of the population is considered to be either *susceptible* (S) if they have not yet had the virus, *exposed* (E) if they have come into contact with others who have had the virus and are incubating the virus but not yet contagious, *infectious* (I) if they have the virus and are contagious, or *removed* (R) if they have either recovered or have died from the virus. Individual members of the population either remain in the current SEIR state or progress to the next stage at each computational timestep. Unlike SIR models [17, 18], the exposed state was included because the incubation period of COVID-19 creates a time lag between being exposed and becoming infected [19, 20]. Evidence also suggests that individuals who have recovered from COVID-19 are immune to future infection, at least temporarily [21]. We do not distinguish between the spectrum of asymptomatic to severe infections because the lack of data on infection severity would make calibration difficult. In our data, many asymptomatic individuals with potential exposure were tested for COVID-19. Viral spread parameter values are calibrated for each county due to regional differences in population density and social activity that influence transmission. We consider five distinct time periods that are based on state-level policies such as stay-at-home orders and business restrictions. The number of individuals in each stage of the virus per region and per day is defined by a set of difference equations, governed by both calibrated and exogenous parameters. Calibration was achieved via gradient descent.

The contributions of this paper are as follows. We propose a novel network-based SEIR model that includes the spread of infections through short-term travel. During the calibration process, we also aim to predict the infection detection probability. We use this model to demonstrate the effects of travel on COVID-19 spread through a case study in Minnesota. Results show the predicted spread of COVID-19 between March 22 and June 22, 2020. We analyze the effects of different travel scenarios on infections, and present the calibrated parameters. Finally, we predict future infections through December 31, 2020 under several travel and rate of spread scenarios.

## Methodology

We propose a network-based SEIR model augmented with short-term travel exposure by infected individuals in other nodes. SEIR and similar models have been extensively used to study outbreaks of infectious diseases [10–12], including COVID-19 [13–16]. Consider a network G=(N,A) where N is the set of nodes and A is the set of links between nodes. In this model, short-term travel represents travel between nodes within a single day, and long-term travel represents individuals who remain at a different node.

### Short-term travel model

In the short-term SEIR travel model, infectious individuals at node *i* may expose people in *j* through travel, but are modeled as returning to node *i* at the end of the day. Let *S*_*i*_(*t*), *E*_*i*_(*t*), *I*_*i*_(*t*), and *R*_*i*_(*t*) represent the number of susceptible, exposed, infectious, and removed individuals at node *i* at time *t*. The population at node *i* is *N*_*i*_(*t*) = *S*_*i*_(*t*) + *E*_*i*_(*t*) + *I*_*i*_(*t*) + *R*_*i*_(*t*). The dynamics of the proposed model are given as follows:

$$S_{i}\left(t+1\right)=S_{i}\left(t\right)-\rho_{i}\left(t\right)S_{i}\left(t\right)\frac{I_{i}\left(t\right)}{N_{i}\left(t\right)}-\sum_{j\in N}\xi \rho_{i}\left(t\right)S_{i}\left(t\right)\frac{\mu_{ji}\left(t\right)I_{j}\left(t\right)}{N_{j}\left(t\right)}$$ (1)

$$E_{i}\left(t+1\right)=E_{i}\left(t\right)+\rho_{i}\left(t\right)S_{i}\left(t\right)\frac{I_{i}\left(t\right)}{N_{i}\left(t\right)}-\frac{1}{\sigma }E_{i}\left(t\right)+\sum_{j\in N}\xi \rho_{i}\left(t\right)S_{i}\left(t\right)\frac{\mu_{ji}\left(t\right)I_{j}\left(t\right)}{N_{j}\left(t\right)}$$ (2)

$$I_{i}\left(t+1\right)=I_{i}\left(t\right)+\frac{1}{\sigma }E_{i}\left(t\right)-\frac{1}{\ell }I_{i}\left(t\right)$$ (3)

$$R_{i}\left(t+1\right)=R_{i}\left(t\right)+\frac{1}{\ell }I_{i}\left(t\right)$$ (4)

where *σ* is the average incubation time, *ℓ* is the average time until recovery or death, *ρ*_*i*_(*t*) ≥ 0 is the rate of spread per day at node *i* at time *t*, *μ*_*ij*_(*t*) is the proportion of individuals at node *i* traveling to *j* at time *t*, and *ξ* ∈ [0, 1] is the reduced probability of travel for infectious individuals. *μ*_*ii*_(*t*) = 0 because intra-county spread within county *i* is accounted for using the term $\rho_{i}\left(t\right)S_{i}\left(t\right)\frac{I_{i}\left(t\right)}{N_{i}\left(t\right)}$. *μ*_*ij*_(*t*) is less than 1 because not all individuals in *i* will travel to *j*. *μ*_*ij*_(*t*) can include travel by any mode. *ξ* is included because symptomatic individuals are less likely to travel. This model assumes that all exposed individuals eventually become infectious, which is not true in reality. The observed number of infections does not include exposed individuals who do not become infectious. When calibrating the model against the observed number of infections, the parameter *ρ*_*i*_(*t*) would be reduced, which models such individuals as not becoming exposed. Future work could expand the SEIR states and introduce more complex transitions, but the purpose of this paper is to relate COVID-19 spread to short-term travel.

The reproductive number *r*_*i*_(*t*) is found by taking the number of new infections divided by the number of infectious individuals at that node, multiplied by the duration of infection *ℓ*:

$$\begin{array}{c} r_{i}\left(t\right)=\frac{\rho_{i}\left(t\right)S_{i}\left(t\right)\frac{I_{i}\left(t\right)}{N_{i}\left(t\right)}+\sum_{j\in N}\xi \rho_{i}\left(t\right)S_{i}\left(t\right)\frac{\mu_{ji}\left(t\right)I_{j}\left(t\right)}{N_{j}\left(t\right)}}{I_{i}\left(t\right)+\sum_{j\in N}\xi \mu_{ji}\left(t\right)I_{j}\left(t\right)}\ell \end{array}$$ (5)

When calibrating the model, we acknowledge that only a fraction of infections were detected. Let λ_*i*_(*t*) be the proportion of infections that are detected. We calibrate λ_*i*_(*t*), *E*_*i*_(0), *ρ*_*i*_(*t*), and *ξ* against reported infections ${\hat{I}}_{i}\left(t\right)$ by solving the problem

$$\begin{array}{cccc} & \text{min} & & Z=\sum_{t=0}^{T}\sum_{i\in N}{\left(I_{i}\left(t\right)\lambda_{i}\left(t\right)-{\hat{I}}_{i}\left(t\right)\right)}^{2} \end{array}$$ (6)

s.t. (1)–(4)

Values for *σ* and *ℓ* are taken from the literature. The objective function is the sum of squared errors in predicted infections. To avoid overfitting, we define time intervals *π* for which *ρ*_*i*_(*t*) and λ_*i*_(*t*) are constant, respectively. We impose the additional constraint that *ρ*_*i*_(*t*) is constant for all time steps *t* ∈ *π*, for all time intervals *π* ∈ Π_*ρ*_. Similarly, λ_*i*_(*t*) is constant for all *t* ∈ *π* for intervals *π* ∈ Π_λ_. The sets of intervals Π_*ρ*_ and Π_λ_ are chosen to reduce overfitting. In the results, Π_*ρ*_ was chosen based on social distancing or lockdown events, and one λ_*i*_(*t*) value was used per week. In other words, *ρ*_*i*_(*t*) and λ_*i*_(*t*) are piecewise constant variables.

The calibration problem is likely not convex due to the cubic term $\rho_{i}\left(t\right)S_{i}\left(t\right)\frac{I_{i}\left(t\right)}{N_{i}\left(t\right)}$ in (1). *ρ*_*i*_(*t*) is a decision variable, and *S*_*i*_(*t*) and *I*_*i*_(*t*) are functions of decision variables. Nevertheless, we apply gradient descent methods to find a local minimum. One important novel feature is the calibration of λ_*i*_(*t*) which predicts the proportion of infections that are detected in addition to reproductive number and number of infectious individuals.

## Data

The daily number of reported infections per county was obtained from the Center for Systems Science and Engineering at Johns Hopkins University [22]. Reported infections includes both symptomatic and asymptomatic individuals. We used parameters of *σ* = 6.4 and *ℓ* = 7 from [20] for the average incubation and recovery time. Of course, with so little known about COVID-19, these parameters may not be completely accurate.

**Fig 2:**
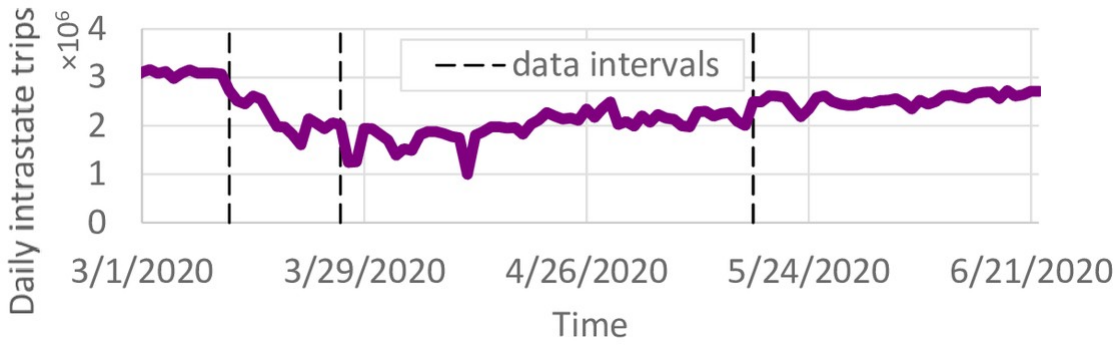
Total county-to-county trips per day.

Daily county-to-county trips were obtained through StreetLight (www.streetlightdata.com/) for the state of Minnesota. StreetLight is a transportation data company providing travel data based on mobile phone location records. These data are based on mobile phone records and represent an estimate of every trip that occurred in Minnesota during the specified time periods. Due to data processing limitations, we obtained four periods of county-to-county travel. The first period, from January 1, 2020 to March 12, 2020, is representative of pre-pandemic travel. From March 13, 2020 to May 17, 2020, working from home became normal and lockdowns were implemented. The final period of data obtained is from May 18, 2020 through May 31, 2020. Data after May 31, 2020 was not available, so the county-to-county travel for the period of March 15, 2020 through May 31, 2020 was used until the end of the time horizon. This may differ somewhat from the actual travel in June. The number of trips per day was further adjusted by the daily vehicle miles traveled recorded in the Minneapolis/St. Paul metropolitan region. The resulting number of intrastate trips per day is shown in Fig 2.

**Fig 3:**
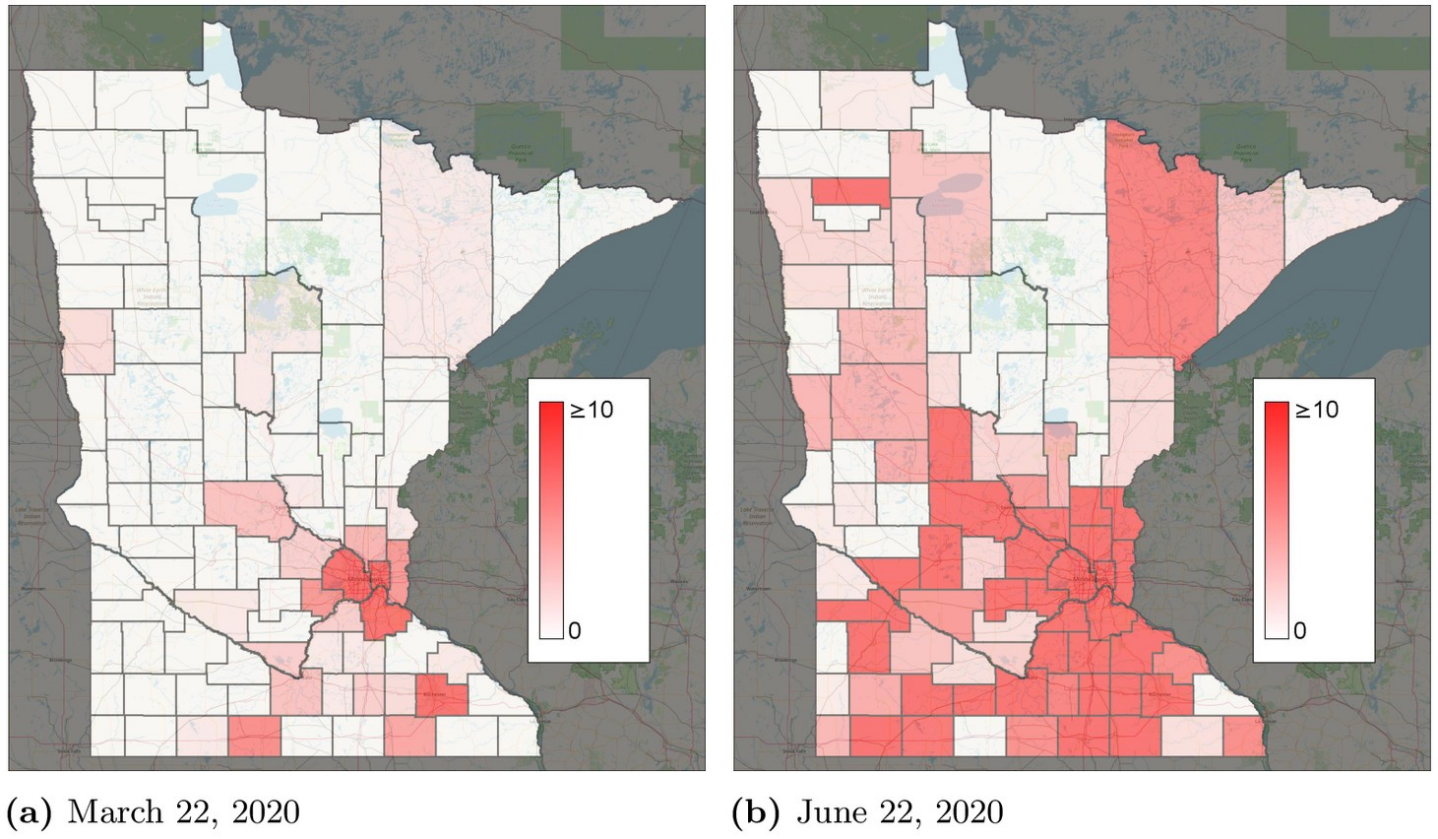
Reported infections by county. (a) March 22, 2020. (b) June 22, 2020.

March 22, 2020 was chosen as the start date because the number of infections was relatively low, yet present. If an earlier start date were chosen, where infections were still primarily spreading from travel from outside of Minnesota, calibrating the model would be difficult due to limited data on interstate and international travel. March 22 is late enough in the pandemic that global travel restrictions were in place, yet early enough that Minnesota had few reported cases in most counties. Consequently, most spread of COVID-19 through Minnesota is expected to occur through intrastate travel or exposures within the same zone. Fig 3a shows the initial reported infections in each county on March 22. Besides Minneapolis/St. Paul and Rochester, very few infections were reported. Most counties have 0 or close to 0 infections. Infections were estimated through June 22, 2020. In contrast to the initial time of March 22, 2020, at the end date of the time horizon, infections have spread across much of the state. Therefore, this starting infection pattern is useful because it tests the ability for the models to predict the spread of COVID-19 through intrastate travel.

## Results

**Fig 4:**
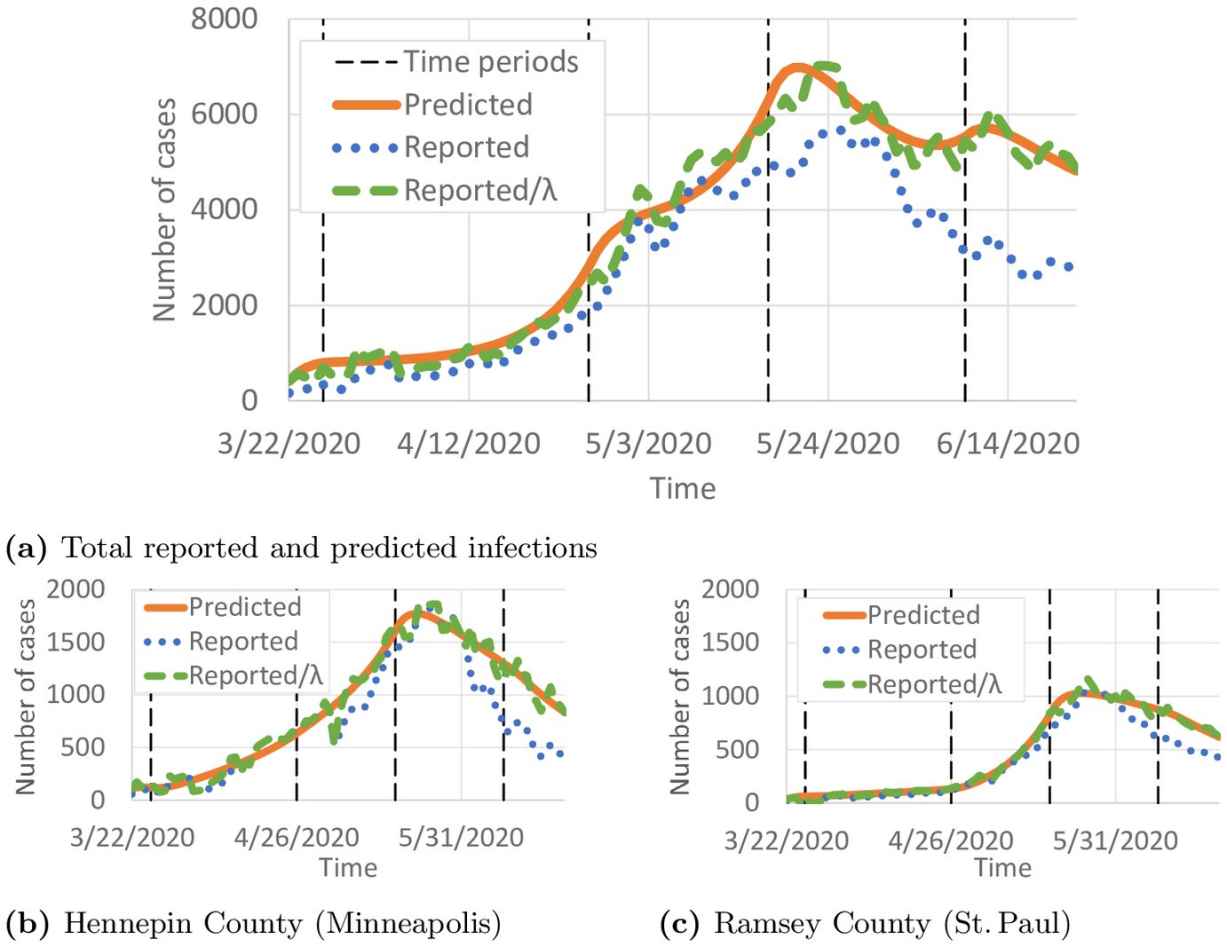
Comparison of predicted and reported infected individuals. Black vertical lines show the intervals in ∏_*ρ*_. (a) Total reported and predicted infections. (b) Hennepin County (Minneapolis). (c) Ramsey County (St. Paul).

We present results from the proposed short-term travel model. Fig 4a compares the total numbers of predicted, reported, and reported cases scaled by the detection probability λ_*i*_(*t*). Overall, the total number of predicted cases seems to be a good fit for $\sum_{i\in N}\frac{{\hat{I}}_{i}\left(t\right)}{\lambda_{i}\left(t\right)}$. Given the COVID-19 testing availability in Minnesota, the differences between $\sum_{i\in N}\hat{I}\left(t\right)$ and $\sum_{i\in N}\frac{{\hat{I}}_{i}\left(t\right)}{\lambda_{i}\left(t\right)}$ seem quite reasonable. The model appears to be generally a good predictor of the variation in cases for individual counties as well. For example, Fig 4b and 4c compare the reported ${\hat{I}}_{i}\left(t\right)$, ${\hat{I}}_{i}\left(t\right)\lambda_{i}\left(t\right)$, and the predicted *I*_*i*_(*t*) for Hennepin and Ramsey Counties, which correspond to the major cities of Minneapolis and St. Paul. Fig 3 and S1 Video. illustrate the predicted spread of COVID-19 across Minnesota through intrastate travel over time.

### Effects of travel on COVID-19 spread

We now demonstrate the importance of intrastate travel on COVID-19 spread. Using county-to-county travel data from Minnesota, as well as observed reduction in vehicle miles traveled, we estimated the number of daily trips between every pair of counties (Fig 1) and calibrated the SEIR model accordingly. The number of daily trips from *i* to *j*, *τ*_*ij*_(*t*) is converted to *μ*_*ij*_(*t*) via

$$\begin{array}{c} \mu_{ij}\left(t\right)=\frac{\tau_{ij}\left(t\right)}{N_{i}\left(t\right)} \end{array}$$ (7)

Overall, the average number of daily county-to-county trips reduced from 3.03m prior to March 12 to 2.19m then to 1.74m after March 26. However, there were still many daily county-to-county trips. The average number of daily trips started to increase again in late April.

**Fig 5:**
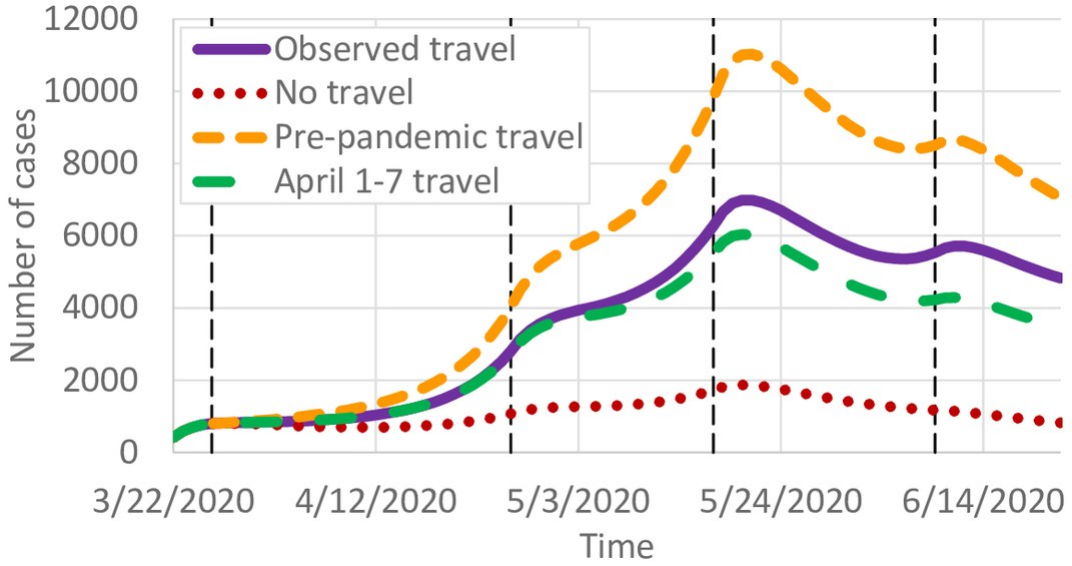
Effects of travel on COVID-19 infections.

**Fig 6:**
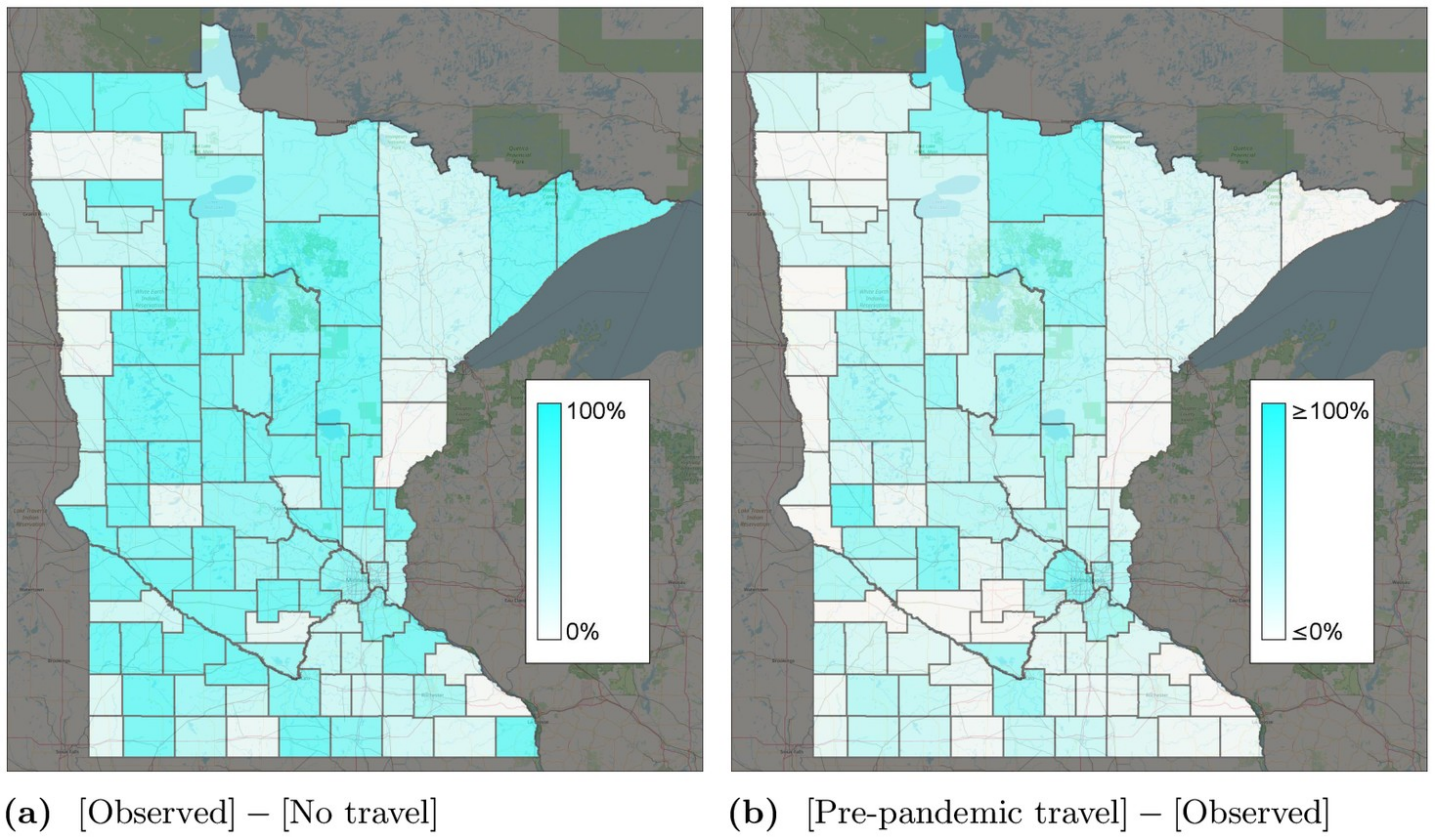
Percent change in infections for different travel scenarios. The “observed” scenario is the base travel scenario. (a) [Observed]—[No travel]. (b) [Pre-pandemic travel]—[Observed].

In the calibrated model, 22.1% of predicted infections were caused by infectious individuals traveling to other counties. Fig 5 shows the total predicted infections when all county-to-county travel is removed, with all other parameters kept constant. Because of the exponential growth in infections, travel reductions result in major differences in COVID-19 spread. We also study the county-specific cumulative number of cases $\Sigma I_{i}=\frac{1}{\sigma }\sum_{t=0}^{T}I_{i}\left(t\right)$, found by taking the sum of all cases over time, and dividing by the case duration *σ*. The greatest change in the number of cases occurred in and around the Minneapolis/St. Paul metropolitan region. This may be due to high daily travel between these counties which includes some daily commuting trips. It could also be due to a higher population density or other characteristics. Fig 6a shows the percent of cases between March 22, 2020 and June 22, 2020 resulting from travel. With no travel, far fewer infections are reported, not only in rural counties, but also in the suburban counties around Minneapolis/St. Paul.

Fig 6b shows the percent change in infections if travel remained at 12.7m trips per day instead of reducing as observed. Fig 5 predicts a significant increase in the number of cases, from around 7,000 to 11,000 at the peak. In contrast, if travel remained at April 1–7 levels (Fig 1) instead of gradually increasing, we predict slightly fewer infections. Fig 5 shows the county-specific percent changes in cumulative infections. These results suggest that reductions in intrastate county due to work-from-home and other lockdown policies had a significant effect on reducing COVID-19 spread. As travel started to increase again, infections correspondingly increased as well.

As part of the model calibration, we estimated the parameter *ξ*, the probability that a person who is infectious will continue their normal county-to-county travel. The calibrated value of *ξ* was 31.0%, which is not dissimilar to reported asymptomatic proportions. For instance, [23] estimated that 17.9% of infections were asymptomatic.

### Calibrated parameters

We analyze the reproductive number and infection detection probability parameters of the calibrated model. To avoid overfitting, only 5 different rate of spread parameters *ρ*_*i*_(*t*) were used per county. These periods span the intervals between the dates March 22, March 27, April 27, May 18, June 10, and June 22, 2020, which were chosen based on COVID-19 policy changes from the Minnesota government. Different detection probability parameters per county were also used due to the possibility of different testing availability and individual behaviors in different counties. For instance, testing might have been less available in certain rural counties. We used a single detection probability per week per county to reduce overfitting. If data on the number of undetected COVID-19 infections were available, we could compare our predicted detection probabilities with such data to check whether overfitting occurs. Unfortunately, these data are not available, and therefore, we cannot be sure to what extent overfitting may be occurring.

**Fig 7:**
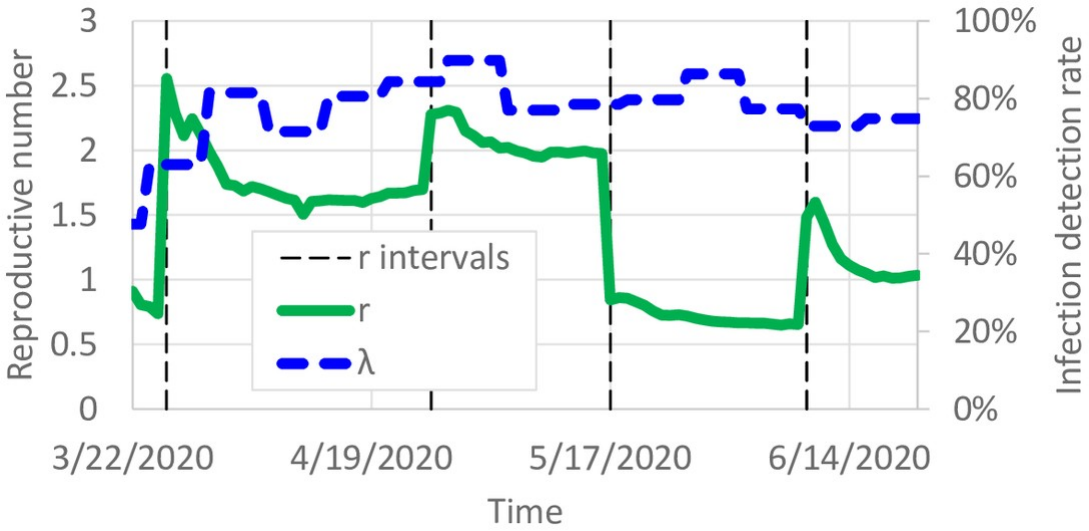
Calibrated reproductive number and detection probability.

**Fig 8:**
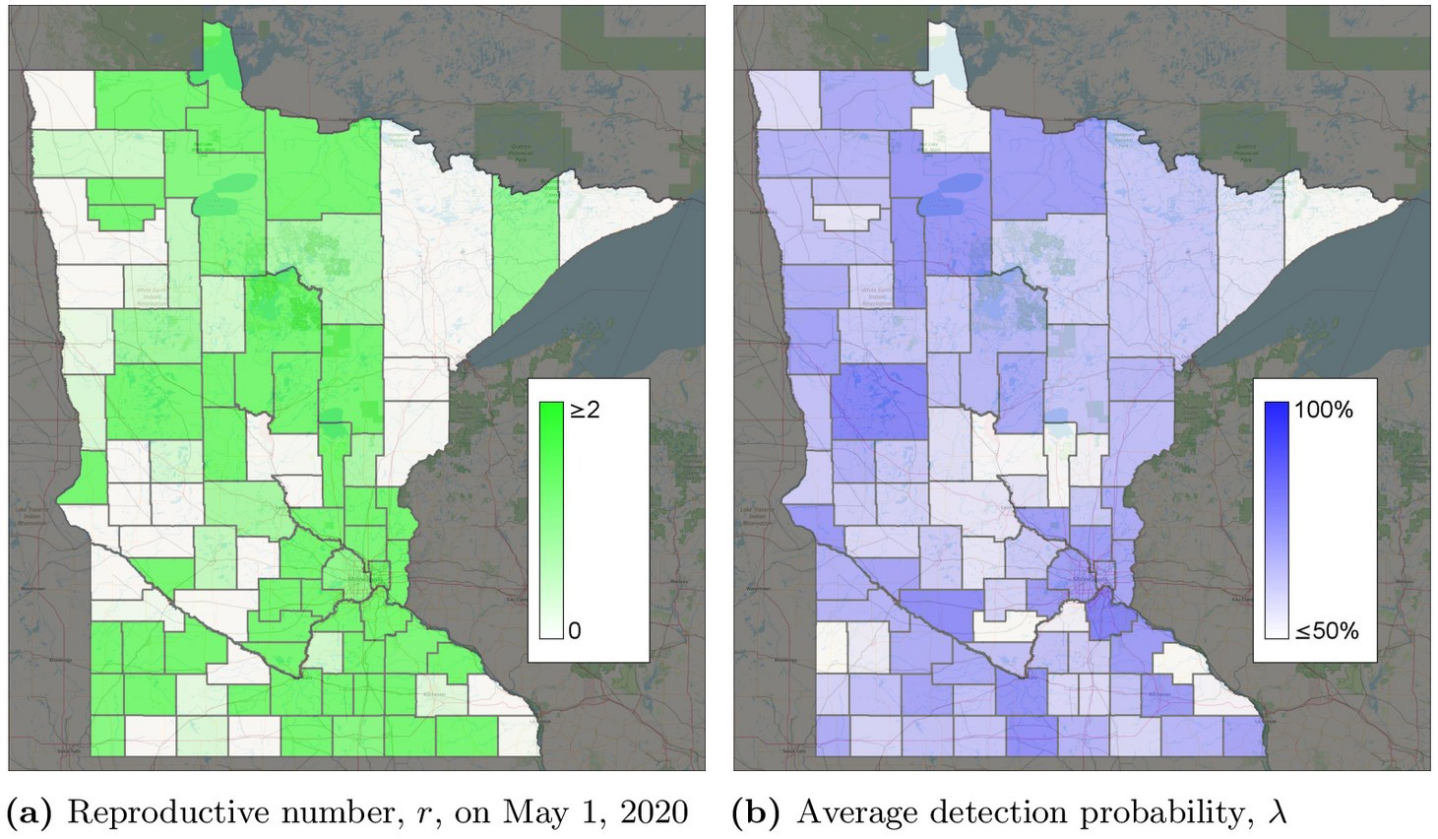
Calibrated parameters by county (see S1 Video for more detail). (a) Reproductive number, *r*, on May 1, 2020. (b) Average detection probability, λ.

The calibrated model predicts the daily reproductive number, i.e. the number of infections per additional infection. We calibrated the model with different probabilities of infection for each of several intervals corresponding to policy events. We plot the estimated average reproductive number $\bar{r}\left(t\right)$, weighted by county population, in Fig 7. Note that although only 5 rate of spread variables were used, the reproductive number varies each time step because it also depends on the number of susceptible individuals in the population. The initial low value results from the low overall number of cases, but the average reproductive number soon jumped to almost 3 per infection. On March 27, 2020, the stay-at-home order started, and the reproductive number was observed to decrease to around 1.5. After some workplaces reopened on April 27, the calibrated reproductive number jumps to around 2 before decreasing in May. However, when businesses and restaurants started reopening on June 10, the reproductive number increased to around 1. Overall, these reproductive numbers correspond to the change in cases shown in Fig 4a. Fig 8a shows reproductive numbers per county on May 1, 2020. High COVID-19 reproduction was observed in the 7 counties around Minneapolis/St. Paul, as well as in some of the rural counties around the state. The different reproductive numbers per county can be explained by different behaviors throughout the state. In rural counties, social distancing may be easier than in urban or suburban counties. Indeed, the reproductive number seems high around major cities such as Minneapolis/St. Paul.

Fig 7 also shows the predicted average detection probability $\bar{\lambda }\left(t\right)$, also weighted by county population. Overall, the average detection probability was initially around 50%, which gradually increased to around 75%–80% of cases. To avoid overfitting, a single λ_*i*_(*t*) value was estimated per week. Although it is difficult to predict the number of undetected infections from only the reported infections, asymptomatic transmission is a known characteristic of COVID-19 [24]. Mild or asymptomatic infections that remain undetected would still cause transmission to others, which would be detected at a probability of λ_*i*_(*t*). In other words, the balance between the exponential growth of infections at the reproductive number and the linear detection probability λ_*i*_(*t*) admits calibration. For instance, if λ_*i*_(*t*) was incorrectly estimated to be 1, then the growth in the number of infections over subsequent days could lead to a higher error in calibration objective (6).

Without extensive testing of the general population, the true detection probability is unknown in most datasets which makes an accuracy comparison difficult. However, considering the observation of [23] of 17.9% infections being asymptomatic, a 75%–80% detection probability is reasonable and can easily include individuals with asymptomatic or mild cases. Testing was available for symptomatic individuals in Minnesota, as well as asymptomatic individuals working in medical care. The calibrated detection probabilities also improved how well the model fit the data. λ_*i*_(*t*) = 1 for several nodes and time periods, suggesting that if λ_*i*_(*t*) = 1 were the best fit, it would have been chosen. Fig 8b shows the average detection probability per county. Estimated detection probabilities seem to be higher in Minneapolis/St. Paul than some of the surrounding suburban counties. This could be due to different behaviors or characteristics of people, such as people in urban areas having more comorbidities resulting in more severe cases.

### Sensitivity analyses

**Fig 9:**
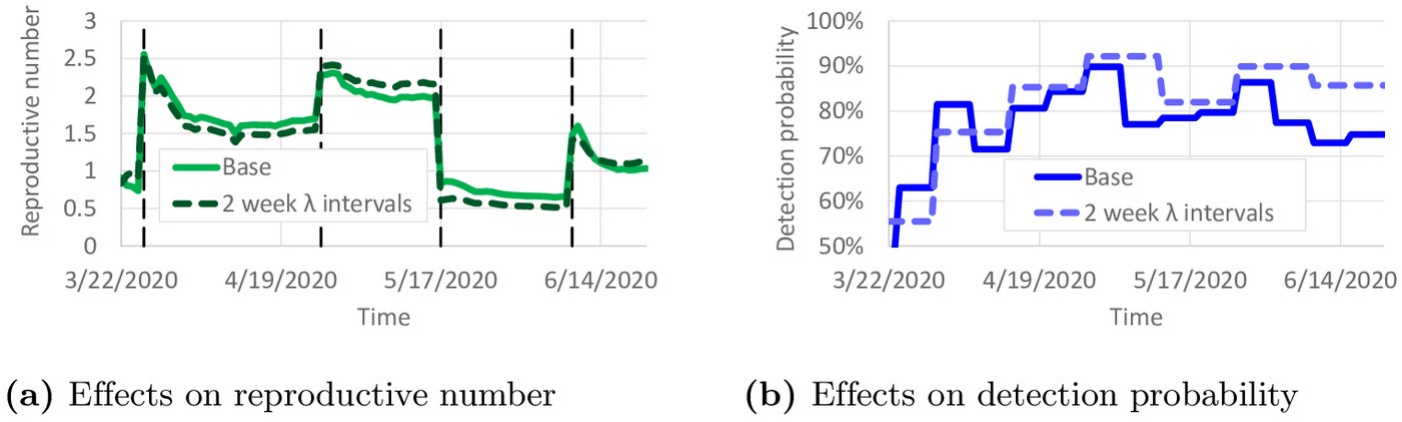
Effect of 2-week λ intervals on calibrated parameters. (a) Effects on reproductive number. (b) Effects on detection probability.

Overfitting is a potential issue with this model calibration. To reduce the impact of overfitting, a constant value of λ_*i*_(*t*) was used for 1-week intervals in most of the results. Here, we study the impact of overfitting by using a constant value of λ_*i*_(*t*) for 2-week intervals. We calibrated all model parameters again using these new λ intervals. Fig 9 compares the calibrated parameters with the “base” case of 1-week λ intervals. Only small variations in *r*_*i*_(*t*) are observed in Fig 9a. Larger variations in λ_*i*_(*t*) are shown in Fig 9b, but overall λ_*i*_(*t*) remains fairly similar for much of the time horizon. The calibrated value of *ξ* with 2-week λ intervals was 0.307, which is close to *ξ* = 0.310 with 1-week λ intervals. The small variations in *r*_*i*_(*t*) suggest that the predicted number of infectious individuals would not change much.

**Fig 10:**
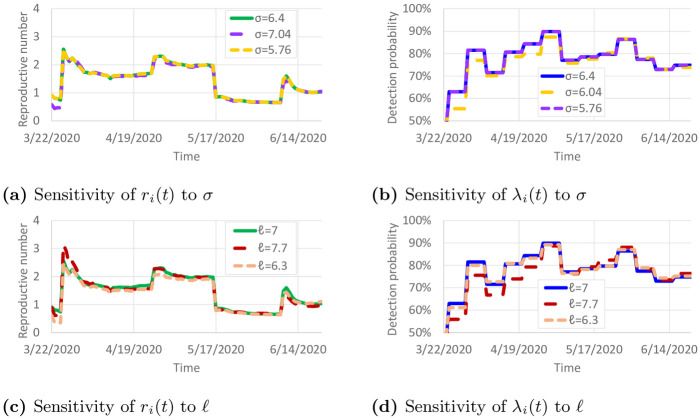
Sensitivity of calibrated parameters to variations in *σ* and *ℓ*. (a) Sensitivity of *r*_*i*_(*t*) to *σ*. (b) Sensitivity of λ_*i*_(*t*) to *σ*. (c) Sensitivity of *r*_*i*_(*t*)(t) to *ℓ*. (d) Sensitivity of λ_*i*_(*t*) to *ℓ*.

Parameter values of *σ* = 6.4 and *ℓ* = 7 were taken from [20]. However, these values are based on early work on COVID-19, and the true values of these parameters could be different. To examine the sensitivity of the results to variations in *σ* and *ℓ*, we recalibrated the model with ±10% variations to *σ* and *ℓ*. The resulting calibrated values of *r*_*i*_(*t*) and λ_*i*_(*t*) are shown in Fig 10. Only small differences are observed in the calibrated parameters. This suggests that the reproductive numbers and detection probabilities are fairly robust against small changes in *σ* and *ℓ*. Table 1 presents the sensitivity of *ξ* with respect to these variations in *σ* and *ℓ*. *ξ* remained fairly similar, except when *ℓ* was increased to 7.7. With *ℓ* = 7.7, infectious individuals remain contagious in the model for 10% longer, so the observed COVID-19 spread could be achieved with less daily travel per infectious individual.

**Table 1:** Sensitivity of *ξ* with respect to *σ* and *ℓ*.

*σ*	*ℓ*	*ξ*
6.4	7	0.310
7.04	7	0.312
5.76	7	0.310
6.4	7.7	0.234
6.4	6.3	0.323

### Projected infections

We use the model to predict the daily number of infected individuals through December 31, 2020 under three travel scenarios: 1) observed county-to-county travel from May and June; 2) travel reductions observed in April (Fig 1); and 3) no travel between counties. We note that since the “observed travel” scenario does not include travel observations past June, it is difficult to project whether travel would remain at summer levels or change in the fall. Pre-pandemic travel was not included because observed travel increased to nearly pre-pandemic levels by the end of June (Fig 1).

**Fig 11:**
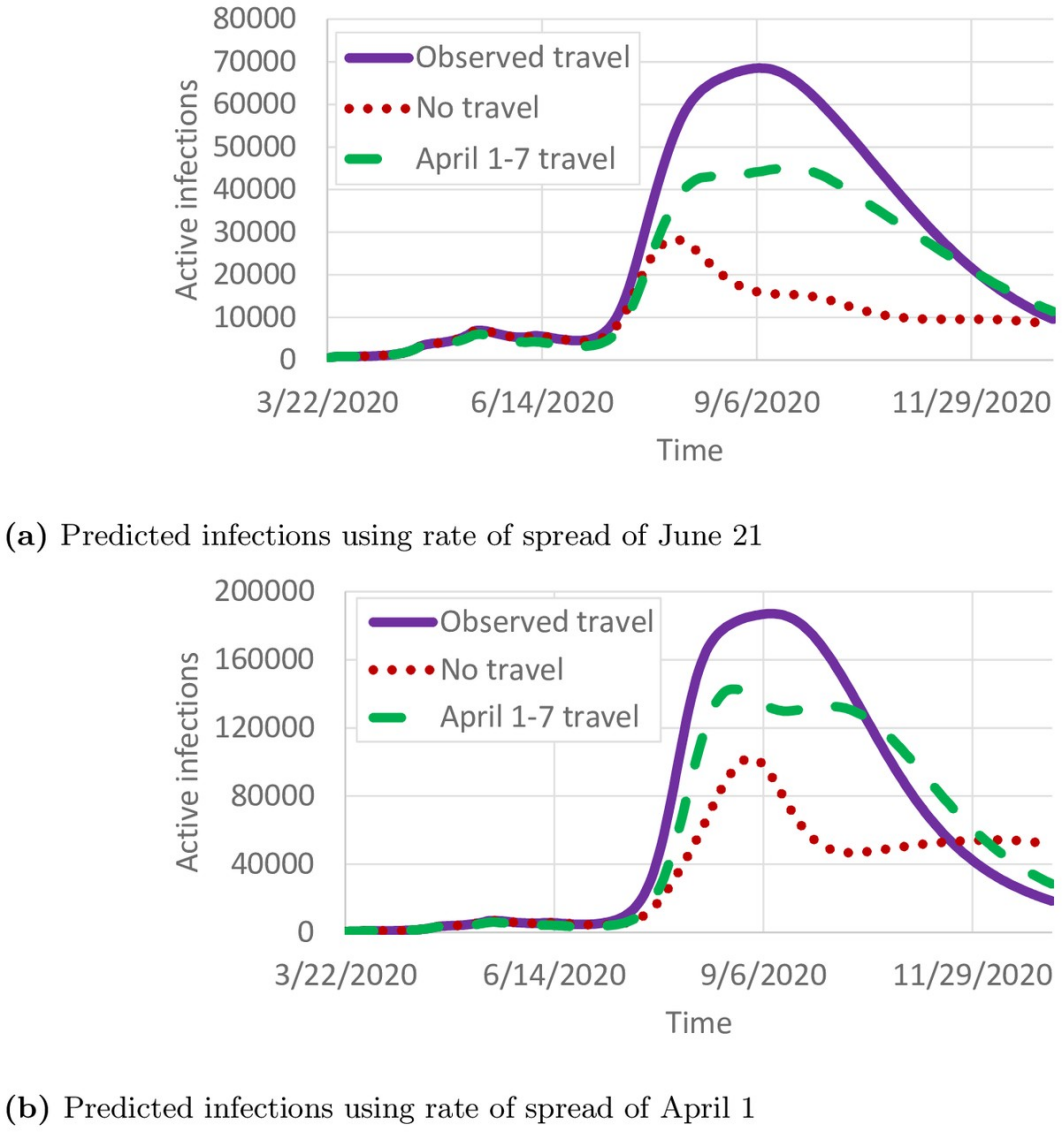
Projected cases. (a) Predicted infections using rate of spread of June 21. (b) Predicted infections using rate of spread of April 1.

Fig 11a shows the number of projected cases using the rate of spread from June 21. Compared with the observed travel scenarios, the no travel scenario fares far better, peaking at 28,082 active infections compared with 68,523 active infections. The travel reductions in April cause a large decrease in the number of infections, though not as large as removing travel entirely. The relatively small peak is primarily due to the smaller reproductive number in late June (Fig 7). By December 31, 2020, 1,104,440 total infections are expected. In contrast, if the rate of spread from April 1 is used, the number of infections is far higher (Fig 11b), peaking at 187,014 active infections on September 9, and with 2,595,072 individuals infected by December 31, 2020. Overall, Fig 11 shows that reducing travel can have a significant impact on the number of cases, but reducing the reproductive number is far more important. For reference, the total population of Minnesota as used in this study is 5,639,589.

### Predicted numbers of removed individuals

**Fig 12:**
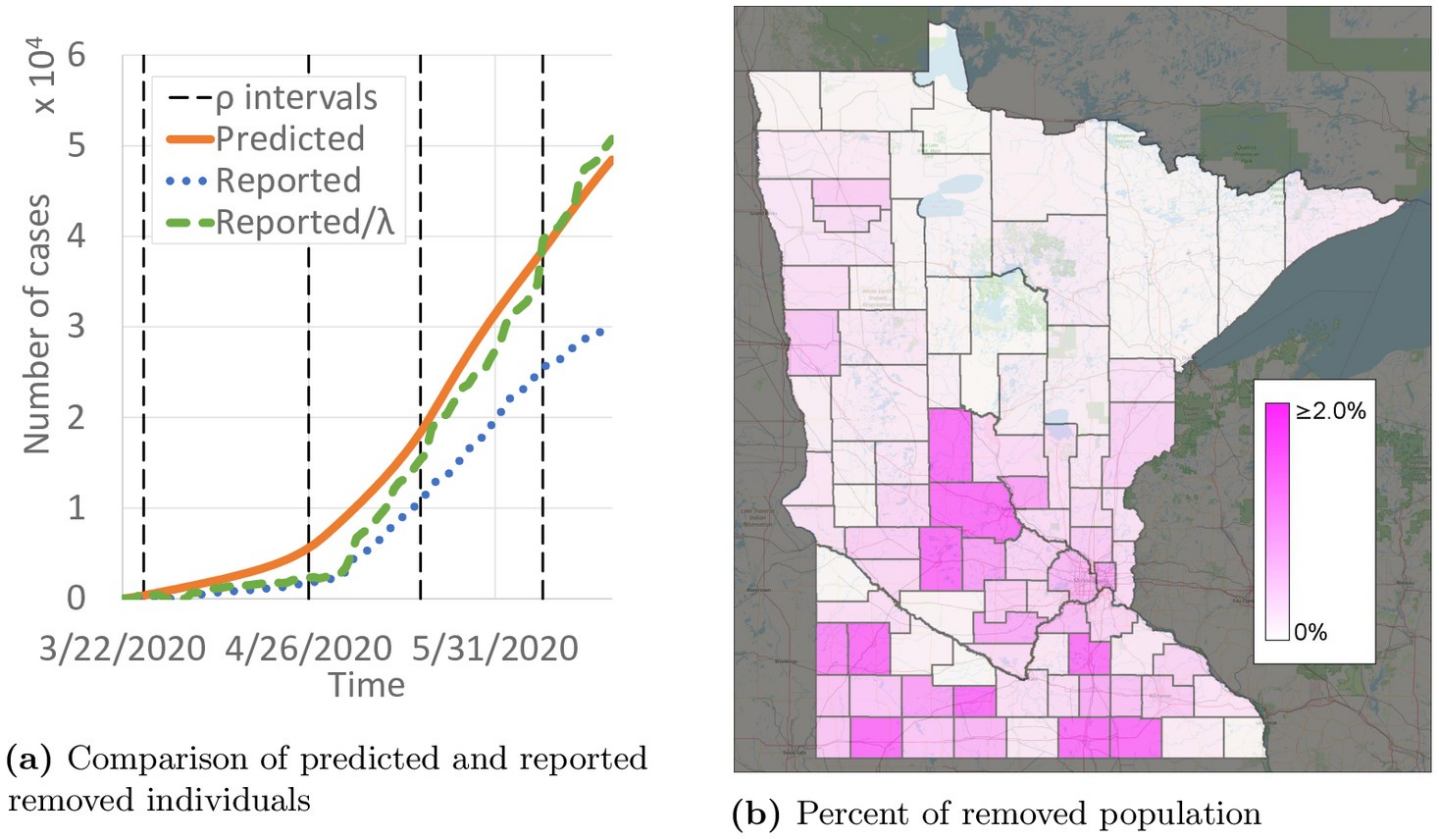
Predictions of removed (either recovered or deceased) individuals. (a) Comparison of predicted and reported removed individuals. (b) Percent of removed population.

People who can no longer become infected due to already having been infected with COVID-19 (or from a vaccine, when one is developed) are counted as removed. Although it is not clear how long immunity lasts [25], we assume that removed individuals remain removed for at least the 12 month duration of the predictions here. Having a large population of removed individuals is vital to returning to pre-pandemic activities. Our data includes reported recoveries and deaths from COVID-19. Fig 12a compares the predicted and reported number of removed individuals. As of June 22, 2020, 30,450 individuals were reported to be removed, and 48,470 individuals were predicted to be removed. With a statewide population of 5.64 million, that corresponds to 0.54% and 0.86% of the population, respectively. The distribution of the removed population is far from uniform, though, as shown in Fig 12b. Several urban and suburban counties around the Minneapolis/St. Paul region have 2% or more of their population predicted to be removed, but that proportion is closer to 0% for many of the more rural counties.

### Long-term vs. short-term travel

We also calibrated a long-term travel model—a networked SEIR model where individuals who travel remain at their destination for multiple days [26]. The long-term and short-term model differed in how they modeled travel behavior. In the long-term model, Eqs (1)–(4) are replaced with

$$S_{i}\left(t+1\right)=S_{i}\left(t\right)-\rho_{i}\left(t\right)S_{i}\left(t\right)\frac{I_{i}\left(t\right)}{N_{i}\left(t\right)}+\sum_{j\in N}(S_{j}\left(t\right)\mu_{ji}\left(t\right)-S_{i}\left(t\right)\mu_{ij}\left(t\right))$$ (8)

$$E_{i}\left(t+1\right)=E_{i}\left(t\right)+\rho_{i}\left(t\right)S_{i}\left(t\right)\frac{I_{i}\left(t\right)}{N_{i}\left(t\right)}-\frac{1}{\sigma }E_{i}\left(t\right)+\sum_{j\in N}(E_{j}\left(t\right)\mu_{ji}\left(t\right)-E_{i}\left(t\right)\mu_{ij}\left(t\right))$$ (9)

$$I_{i}\left(t+1\right)=I_{i}\left(t\right)+\frac{1}{\sigma }E_{i}\left(t\right)-\frac{1}{\ell }I_{i}\left(t\right)+\xi \sum_{j\in N}(I_{j}\left(t\right)\mu_{ji}\left(t\right)-I_{i}\left(t\right)\mu_{ij}\left(t\right))$$ (10)

$$R_{i}\left(t+1\right)=R_{i}\left(t\right)+\frac{1}{\ell }I_{i}\left(t\right)+\sum_{j\in N}(R_{j}\left(t\right)\mu_{ji}\left(t\right)-R_{i}\left(t\right)\mu_{ij}\left(t\right))$$ (11)

Calibration objective function (6), including the detection probability λ_*i*_(*t*), was used for both models. Gradient descent was used to calibrate both models. After 200 iterations, reductions in the objective value were less than 0.1% per iteration. The short-term model had a 23.4% error in predicting infections, where error is defined as $\sum_{i\in N}\sum_{t=0}^{T}\frac{|I_{i}\left(t\right)-{\hat{I}}_{i}\left(t\right)/\lambda_{i}\left(t\right)}{{\hat{I}}_{i}\left(t\right)}$. The long-term model had a 36.9% error in predicting infections. This suggests that a short-term travel model is a better predictor for the spread of COVID-19 from intrastate driving trips. The average computation time per iteration was 104.2s on a desktop computer with an Intel i5–8600k CPU clocked at 3.60 GHz with 16.0 GB of memory, which suggests the method may be scaled to larger networks.

## Conclusions

We proposed and calibrated a novel network SEIR model for the spread of COVID-19 from short-term travel (e.g. daily commute trips). Infectious individuals at node *i* can infect both susceptible individuals at node *i*, and susceptible individuals at node *j*, based on exogenous travel rates from *i* to *j*. This model also includes a calibrated detection probability parameter λ_*i*_(*t*), representing the proportion of infections that are actually detected. These two modifications to the standard SEIR model [9] may be useful for studying COVID-19. Since COVID-19 is known to have large numbers of asymptomatic or mild yet contagious infections [23, 24, 27], substantial numbers of infectious individuals have undetected infections and may travel normally while infectious, spreading COVID-19 to new geographic regions.

A case study in Minnesota, which has substantial county-to-county travel but relatively few counties with interstate or international airline travel, shows the importance of intrastate travel in spreading COVID-19. If travel were removed, the number of cases is predicted to peak at 1,870 compared to the actual 6,982. If travel did not decrease from pre-pandemic levels, that peak is predicted to rise to 11,028. Even returning to April 1–7 travel levels results in a substantial reduction in predicted infections for the remainder of the year. Consequently, we conclude that short-term travel has a significant impact on COVID-19 spread, and efforts to reduce typical daily short-term travel, like commuting, could achieve large reductions in both the number of cases and the geographic spread of COVID-19.

We also demonstrate the potential to predict the infection detection probability through calibrated SEIR models. Because asymptomatic or mild infections are still contagious, estimating the true number of infections may be valuable compared with predicting the spread of COVID-19 only from reported infections. Although extensive testing of the general population [28, 29] is the best way to estimate the detection probability, this calibration method may provide a useful alternative when such testing is unavailable. In addition, including the detection probability may achieve a higher model accuracy than calibrating only against reported infections, which ignores the spread of COVID-19 from undetected infections. Overfitting may lead to inaccuracies when the model is used for forward prediction. We explored overfitting by adjusting the duration of the intervals for which λ_*i*_(*t*) is constant, but it is difficult to verify the accuracy without other sources of data.

Although the results provide novel data on the relationship between short-term travel and COVID-19 spread, there are a number of opportunities for improvement. The incubation and recovery rate parameters were based on [20], but more accurate parameters may later become available after further study. The SEIR model could be extended with more states and transitions to capture the variation in disease progression among individuals. Differences in the population demographics among counties might affect infection characteristics as well. The calibrated model predicts reasonable county-specific detection probabilities, but verifying these against widespread testing of the population would lend further validation if such datasets were available. Our travel data provided only the total number of trips between pairs of counties, but different trip types are likely to affect infection spread differently. For instance, home-to-work trips may result in more person-to-person contact than some recreational travel. Including interstate and international travel which were responsible for the original spread of COVID-19 to Minnesota might further improve the model accuracy.

## Supporting information

S1 Video Evolution of COVID-19 spread over time. (MP4)
